# Premature Pembrolizumab-Induced Pulmonary Problems: A Case of Atypical Early-Onset Checkpoint Inhibitor Pneumonitis

**DOI:** 10.7759/cureus.90734

**Published:** 2025-08-22

**Authors:** Vidya N Mullangi, Vignesh Doraiswamy

**Affiliations:** 1 Internal Medicine, The Ohio State University Wexner Medical Center, Columbus, USA; 2 Hospital Pediatrics, Nationwide Children’s Hospital, Columbus, USA; 3 Internal Medicine-Pediatrics, The Ohio State University Wexner Medical Center, Columbus, USA

**Keywords:** cancer immunotherapy, drug induced pneumonitis, immune check-point inhibitor, immune-related adverse event (irae), non-small cell lung carcinoma (nsclc), pd-1 inhibitors, pembrolizumab side effect

## Abstract

Checkpoint inhibitor pneumonitis (CIP) is a rare but potentially devastating complication of immune checkpoint inhibitor therapy. Due to its nonspecific and variable clinical appearance, CIP can be misdiagnosed or recognized late, resulting in delayed treatment and poor clinical outcomes. We report the case of a 65-year-old female patient with chronic obstructive pulmonary disease (COPD), a significant smoking history, and non-small cell lung carcinoma (NSCLC) who had an atypical early, moderate-to-severe CIP after just two infusions of pembrolizumab. During hospitalization, she demonstrated minimal improvement on empiric treatment for COPD, congestive heart failure (CHF) exacerbation, and pneumonia. On day 9 of hospitalization, computed tomography identified ground-glass opacities in the right posterior lung, prompting escalation of steroid treatment to 2 mg/kg/day to treat CIP. The patient subsequently improved, returned to her baseline oxygen requirements, and was discharged with plans to discontinue pembrolizumab therapy. This case report highlights the atypically early and rapid development of pembrolizumab-induced CIP, demonstrating the need for timely diagnosis and medical intervention. It also raises important questions regarding the continuation of immunotherapy following CIP.

## Introduction

Immune checkpoint inhibitor (ICI) therapy was introduced in 2014 with the FDA approval of anti-programmed death-1 (anti-PD-1) agents pembrolizumab and nivolumab, making a significant leap in the treatment of advanced malignancies [1]. Checkpoints are regulatory proteins found on immune cells and cancer cells that help modulate immune responsiveness. Cancer cells can exploit these checkpoints to avoid the immune system's surveillance. ICI therapies work by blocking these proteins, restoring the immune system's ability to recognize and attack cancer cells. However, this heightened immune activation can lead to autoimmune-like inflammation of healthy tissues [1]. 

Pembrolizumab in particular is widely used in the treatment of melanoma and non-small cell lung carcinoma (NSCLC) [2]. The side effects of pembrolizumab in patients with NSCLC include fatigue (19%), rash (10%), diarrhea (8%), hypothyroidism (7%), and checkpoint inhibitor pneumonitis (CIP) (3%), in which monoclonal antibodies begin to attack and destroy lung parenchyma [3]. 

Based on severity and findings on imaging, CIP is categorized into four grades: Grade 1 (asymptomatic disease), Grade 2 (symptomatic involvement of 25-50% of lung parenchyma), Grade 3 (symptomatic involvement of >50% of lung parenchyma), and Grade 4 (life-threatening respiratory failure often requiring urgent interventions like intubation) [4]. Notably, grades 3 and 4 typically require high-dose glucocorticoid treatment and cessation of pembrolizumab immunotherapy [5,6].

When CIP occurs, it typically presents 2-12 months after initiating immunotherapy, but in some of the earliest reported cases, it occurred within 48 hours of initiation or just after four infusion sessions [7,8]. CIP can be difficult to recognize because its symptoms, including cough, dyspnea, and hypoxemia, closely mimic those of more common conditions like chronic obstructive pulmonary disease (COPD) exacerbation, pneumonia, or heart failure. This overlap in symptoms can delay diagnosis and treatment.

We present a case of new severe CIP in an elderly Black woman who received only two infusions of carboplatin/Taxol/pembrolizumab chemo-immunotherapy over one month for stage III squamous cell carcinoma of the lung.

## Case presentation

A 65-year-old woman presented to the emergency department with fatigue, a nonproductive cough, hypoxemia, and acute worsening of dyspnea. Her past medical history included heart failure with preserved ejection fraction (HFpEF), COPD well controlled on 4 liters per minute (LPM) of long-term home oxygen, a former smoker with 45-pack-year history, and stage III squamous cell carcinoma of the lung status post radiation therapy, with the last session five months before this presentation. She had been undergoing chemotherapy with carboplatin and paclitaxel for six months prior to her presentation and recently received two infusions of pembrolizumab in the month leading to presentation.

On arrival, the patient was febrile (101 F), hypotensive (84/50 mmHg), and dyspneic on 4 LPM oxygen with saturations ranging between 94% and 98%. Venous blood gas (VBG) revealed a respiratory alkalosis (pH 7.46, pCO_2_ 34, pO_2_ 52), suggesting preserved ventilation and reducing the suspicion for COPD exacerbation. Although she did not initially require high-flow oxygen or ICU-level care, her dyspnea progressed during hospitalization, ultimately requiring 9 LPM. 

Physical examination revealed diffuse bilateral inspiratory rhonchi and expiratory wheezing. Notably, there were no signs or symptoms of fluid overload, such as lower-extremity edema, orthopnea, or jugular venous distension. Initial diagnostic workup, including respiratory pathogen panel, blood and urine cultures, was negative for infection.

**Figure 1:**
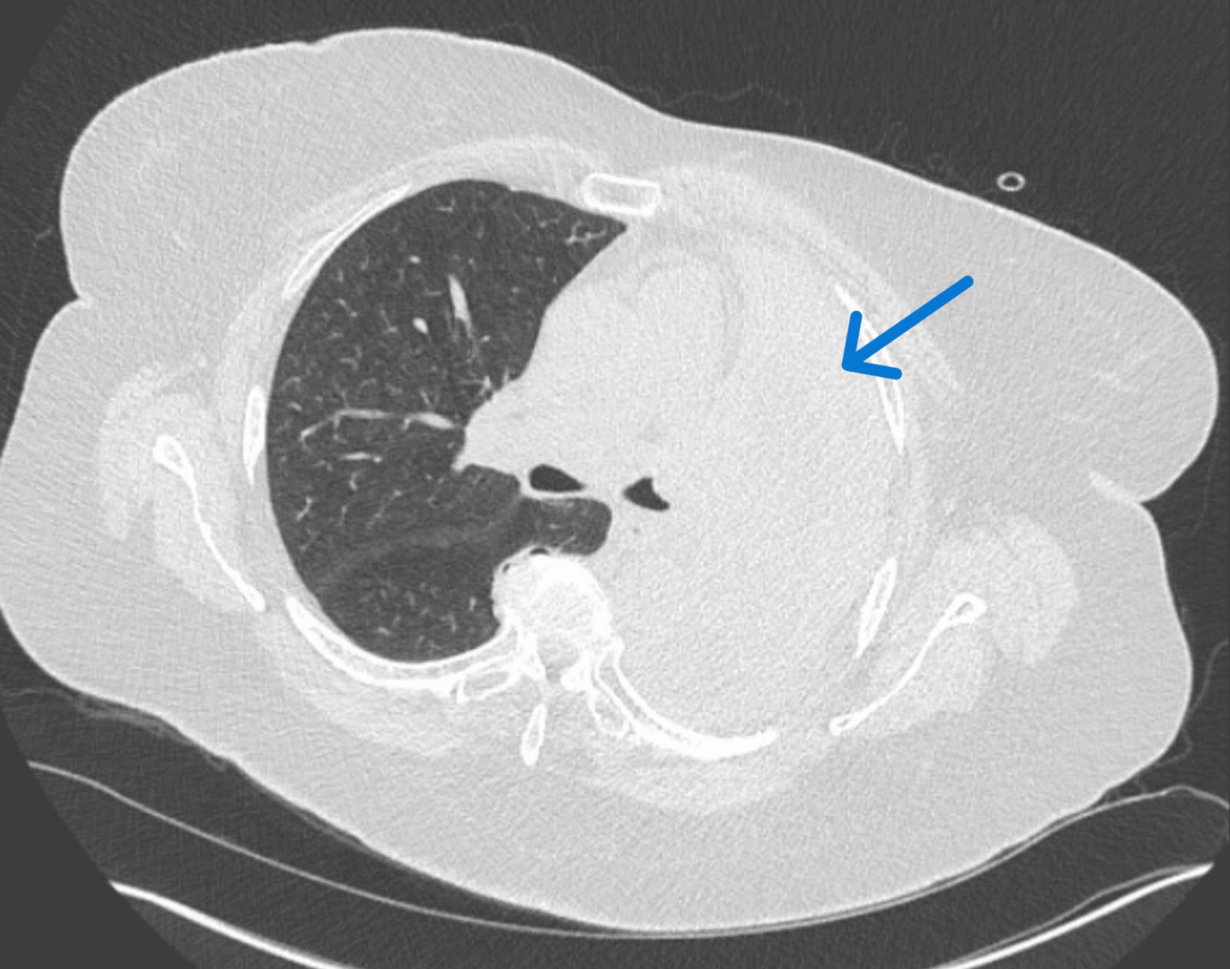
CT Chest Two Weeks Prior to Hospitalization Stable left bronchus occlusion secondary to longstanding squamous cell lung carcinoma (blue arrow).

**Figure 2:**
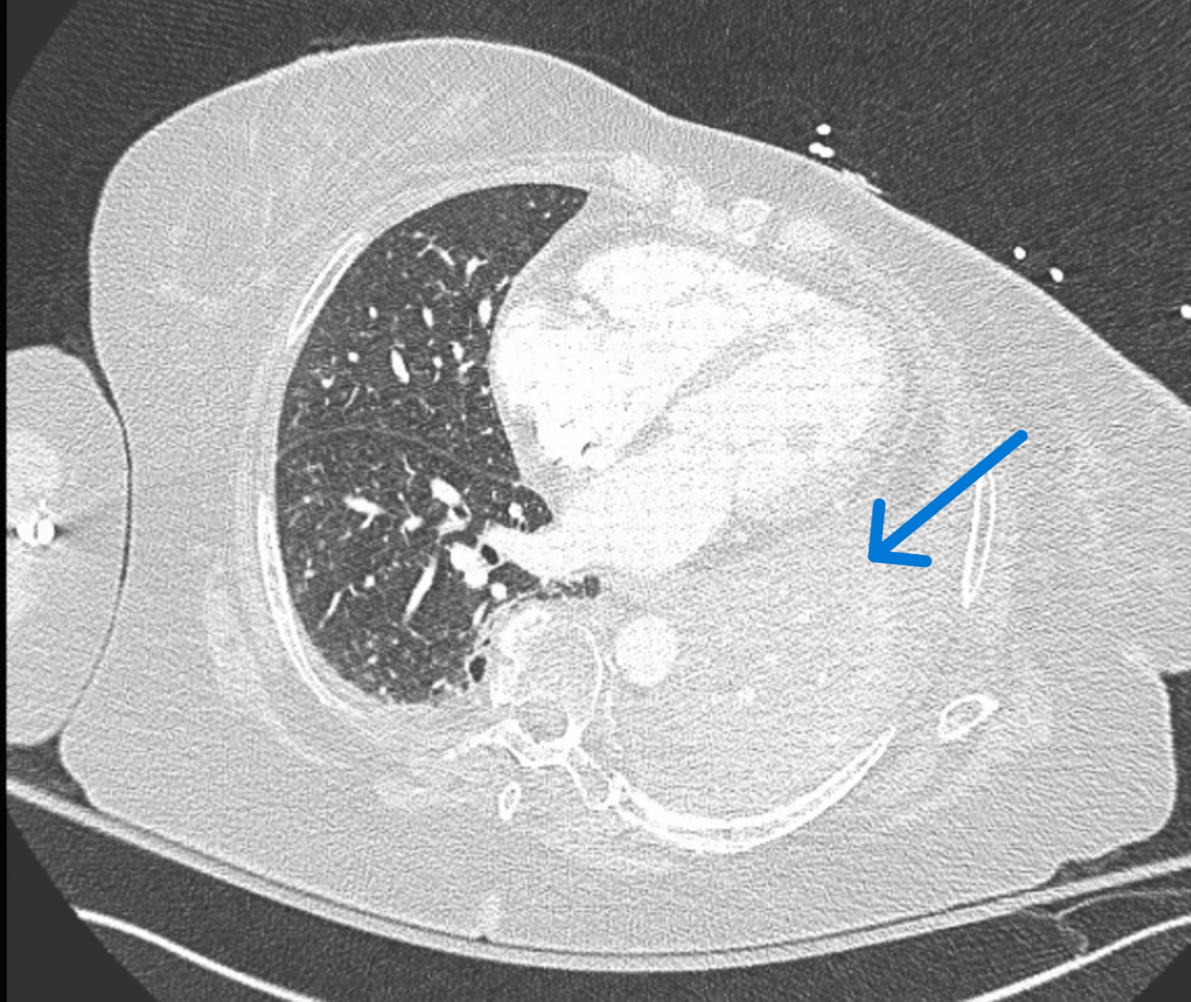
CT PE on Day 1 of Presentation There was no evidence of acute PE. There was stable complete occlusion of L mainstem bronchus and bronchi (blue arrow). PE, pulmonary embolism.

Laboratory studies revealed an elevated brain natriuretic peptide (BNP), chronic anemia with low hemoglobin and low red blood cell count, thrombocytopenia, leukopenia, and elevated lactate (Table 1). Additional blood gases and CRP were not collected during the remainder of her hospitalization. The pancytopenia was likely attributable to recent chemotherapy. The elevated BNP suggested possible heart failure, though the absence of physical examination findings made this less likely. Electrolyte abnormalities included hyponatremia, hypomagnesemia, and hypophosphatemia (Table 1), which were consistent with poor oral intake and chronic illness. Two days prior to admission, she had received 2 units of packed red blood cells for severe anemia presumed to be secondary to her chemotherapy. An initial CT pulmonary angiogram showed a stable left bronchus occlusion secondary to longstanding NSCLC with no new metastases compared to a CT scan from two weeks earlier** **(Figures 1, 2).

**Table 1:** Summary of Laboratory Results at Presentation Electrolyte abnormalities were likely related to poor oral intake and chronic illness. Elevated BNP suggested potential heart failure but no signs of fluid overload were present on examination. Elevated lactate was likely related to tissue hypoxia. Pancytopenia may have resulted from chemotherapy leading to immunosuppression. BNP, brain natriuretic peptide.

Lab	Patient Value	Reference Range (Units)
Sodium (mmol/L)	133	135-145
Magnesium (mg/dL)	1.4	1.6-2.6
Phosphate (mg/dL)	1.9	2.2-4.6
BNP (pg/mL)	755	0-100
Red Blood Cells (M/uL)	2.48	3.91-5.04
White Blood Cells (k/uL)	1.83	3.99-11.19
Platelets (k/uL)	61	150-393
Lactate (mmol/L)	4.4	0.5-1.6
Hemoglobin (g/dL)	7.1	11.4-15.2

Upon admission, the patient required 8-9 LPM of oxygen to maintain saturations ≥92%. The pulmonary team was involved in guiding management, and given the patient's chronic oxygen and persistent dyspnea, the goal was to keep saturations between 94-98%. Due to concern for pneumonia, acute heart failure, and COPD exacerbation, she was started on a seven-day course of empiric IV cefepime, daily furosemide, and daily bronchodilators. One unit of packed RBCs was administered due to chronic worsening of her anemia (hemoglobin 7). On hospital day 4, the pulmonary team started a three-day course of prednisone 60 mg daily. However, the patient demonstrated minimal improvement and remained dyspneic with elevated oxygen requirements (7-9 LPM) to maintain saturations >90%. Steroids were escalated to 1 mg/kg/day for three additional days. During this time, her WBC increased from 8.82 k/µL on hospital day 4 to a peak of 19.72 k/µL on hospital day 9. This leukocytosis was challenging to decipher, as it raised the team's concerns for infection but was also a known effect of corticosteroid therapy. She continued to show minimal clinical improvement despite these measures.

**Figure 3:**
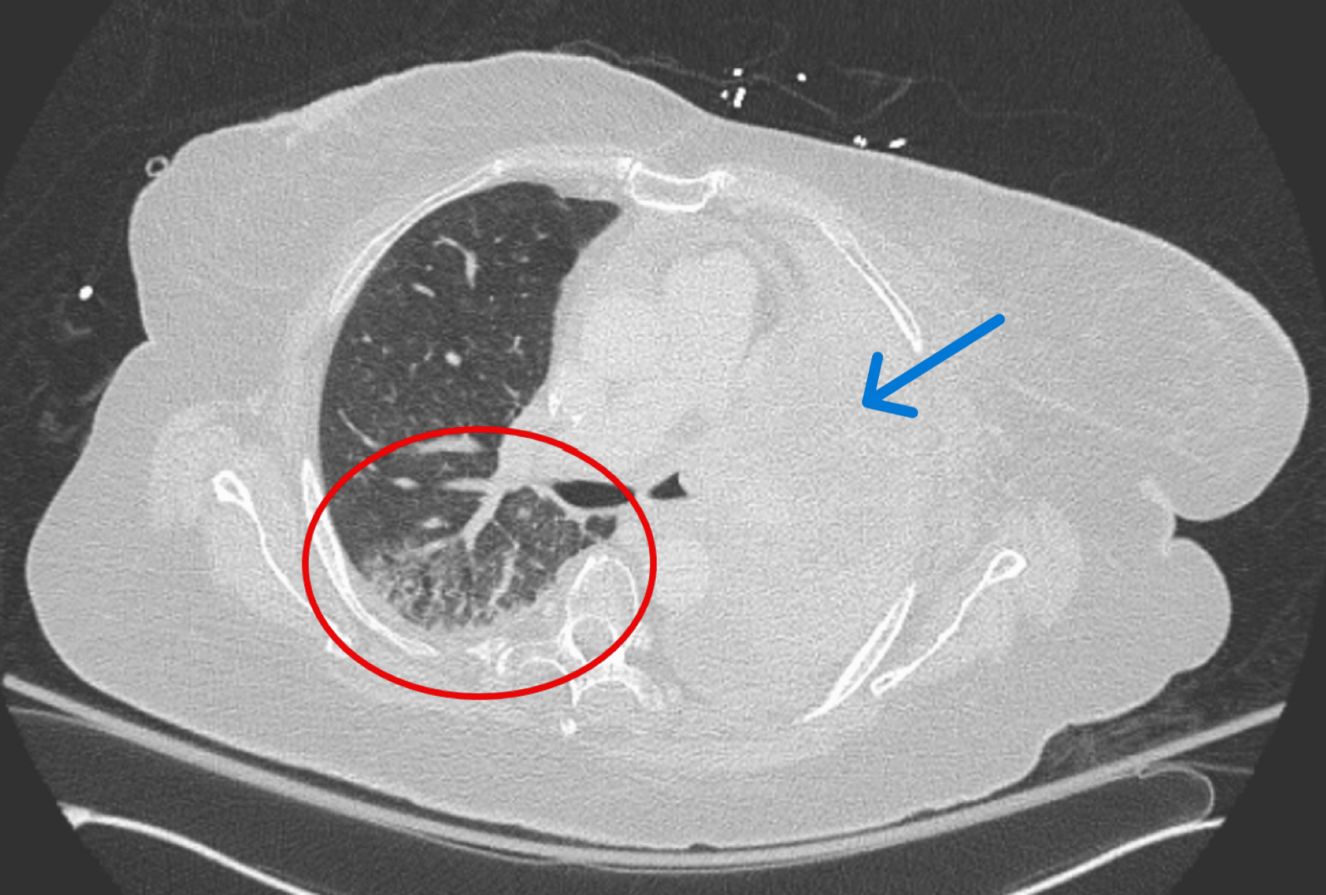
CT Chest on Day 9 of Hospitalization Ground-glass opacities throughout the right lung, most prominent posteriorly (red circle). Stable left bronchus occlusion (blue arrow) with no new foci. These new findings support a diagnosis of checkpoint inhibitor pneumonitis, explaining the patient's worsening respiratory status despite empiric antibiotics, diuretics, and bronchodilators.

On hospital day 9, a repeat CT chest revealed new ground-glass opacities throughout the right lung (Figure 3). Given the patient’s lack of response to treatment for congestive heart failure, COPD, and pneumonia, coupled with the radiographic findings in Figure 3 and recent exposure to pembrolizumab, CIP was considered the most likely diagnosis. High-dose steroids (2 mg/kg/day) and IVIG were initiated. Within three days of this treatment escalation, she showed marked improvement, and oxygen requirements returned to the baseline of 4 LPM of oxygen.

These findings collectively supported the diagnosis of severe CIP. Her CIP was considered to be Grade 3 since, although severe and progressive, it did not result in acute respiratory distress syndrome or require intubation. Several red flags pointed toward this etiology, including a lack of improvement with treatment for other conditions, a negative infectious workup, no radiographic evidence of tumor progression or pulmonary embolism, and the appearance of new ground-glass opacities not present two weeks prior. Most importantly, her clinical course improved only after high-dose corticosteroids and IVIG were initiated. The temporal relationship of her symptoms with ICI therapy, absence of alternative explanations, and steroid responsiveness reinforced the diagnosis of CIP.

**Figure 4:**
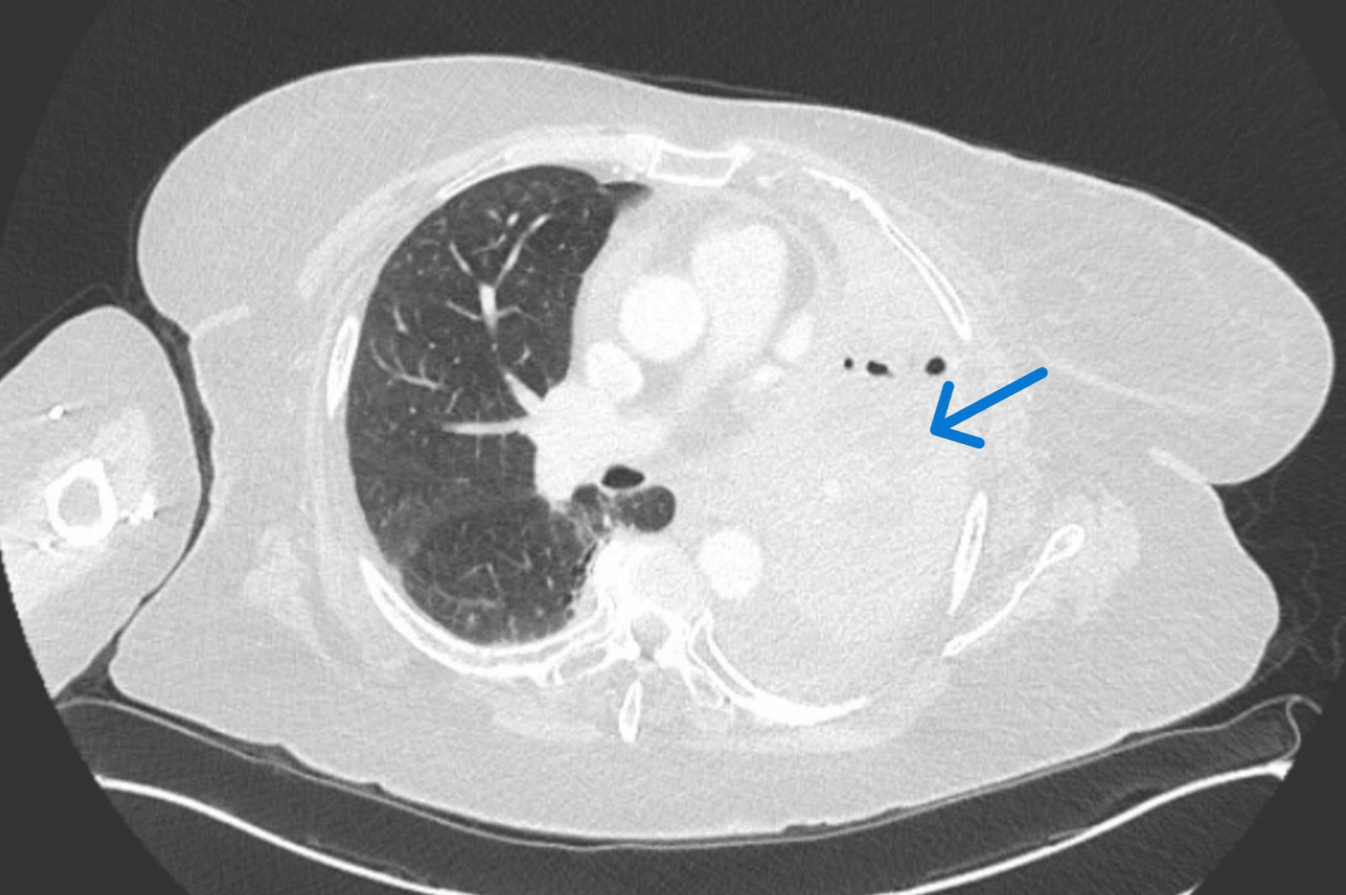
CT Chest After Three weeks of High-Dose Prednisone Treatment Stable left bronchus occlusion (blue arrow) with no new foci. The resolution of ground-glass opacities suggests effective treatment and resolution of checkpoint inhibitor pneumonitis with high-dose steroids and IVIG.

She was discharged to a skilled nursing facility with a prolonged steroid taper and plans for oncology follow-up. At her three-week follow-up visit, a repeat CT scan revealed resolution of ground-glass opacities (Figure 4). Oncology planned to resume chemotherapy without pembrolizumab or enroll the patient in a clinical trial.

## Discussion

CIP is a potentially life-threatening immune-related adverse event (irAE) caused by ICIs, especially those directed against PD-1 and CTLA-4 ligands [5,9]. The pathophysiology is thought to involve a loss of peripheral tolerance, resulting in overactivated T-cells infiltrating and destroying native lung parenchyma, ultimately leading to inflammation, fibrosis, and alveolar injury [3,10-12].

A typical presentation of CIP may include nonspecific symptoms, such as dyspnea, increased oxygen requirements, fatigue, fever, and cough [13]. Because these symptoms overlap significantly with more common pulmonary conditions, CIP is considered a diagnosis of exclusion [13]. Differential diagnoses that should be ruled out include COPD exacerbation, radiotherapy-induced lung injury, transfusion-associated circulatory overload, heart failure, pulmonary embolism, pulmonary edema, infectious pneumonia, and tumor progression [10,13]. In this patient, elevated BNP was concerning for heart failure, but in the absence of physical examination findings, this was lower on the differential. Similarly, COPD exacerbation was less likely given the limited response to bronchodilator therapy and findings on initial VBG. Infectious etiologies were ruled out with respiratory panels and blood cultures, although the rising leukocytosis was confounded by steroid administration. Radiographic evidence, while nonspecific, may aid in the exclusion of alternative etiologies and the diagnosis of CIP. CT imaging may show a variety of findings, ranging from ground-glass opacities or organizing pneumonia to nonspecific interstitial infiltrates [11]. In this patient, CT PE demonstrated no evidence of pulmonary embolism and a stable tumor size without evidence of progression. New right-lung ground-glass opacities seen on repeat imaging were consistent with CIP.

Risk factors for CIP include preexisting lung disease, prior chest radiation, and use of combination immunotherapy, especially anti-PD-1 ligand therapy (particularly when compared to anti-CLTA-4 therapy) [9,14]. Although the incidence of CIP is low, it accounts for approximately 28% of fatal events in patients receiving PD-1 ligand inhibitor therapy, necessitating a high index of suspicion [15,16]. 

The first-line treatment for CIP is high-dose systemic corticosteroids (1-2 mg/kg/day) for 6-8 weeks with a gradual taper. While most patients improve with corticosteroids alone, 15-30% of patients demonstrate a poor response and require additional therapy with IVIG, IL-6 inhibitors, or anti-tumor necrosis factor-alpha (anti-TNFa) agents [6]. Permanent checkpoint inhibitor therapy discontinuation is generally recommended, especially for higher-grade (i.e., 3 or 4) pneumonitis [17].

However, controversy remains regarding the optimal management of ICI therapy following the development of irAEs. Some studies have demonstrated an overall survival benefit in patients with advanced lung cancer and low-grade CIP who were rechallenged with ICI therapy after symptom resolution [18]. Patients with a favorable Eastern Cooperative Oncology Group (ECOG) performance status, a longer duration of initial ICI treatment, and low-grade CIP were associated with improved outcomes following rechallenge [19]. In contrast, poor outcomes and increased risk of irAE recurrence have been associated with high-grade CIP, initial irAE of colitis or hepatitis, advanced age, and elevated levels of IL-6, CRP, WBC, and absolute neutrophil count (ANC) at the time of rechallenge [20]. Therefore, resuming ICI therapy may be a viable option in carefully selected patients and should be considered on an individual basis [20].

This patient’s acute dyspnea, rising oxygen requirements, new imaging findings, and significant response to high-dose steroids alongside lack of clinical improvement with empiric therapy for most other etiologies of respiratory distress were all consistent with pembrolizumab-induced CIP. She had several risk factors for early-onset CIP, including a significant smoking history, prior radiation, and underlying lung disease. When the patient’s CIP developed, it was severe and thus appropriately managed with discontinuation of pembrolizumab and initiation of high-dose steroids and IVIG. Although pembrolizumab-induced CIP is rare, occurring in fewer than 3% of patients, it typically presents between two and 12 months after initiating treatment, with most early cases occurring only after four or more infusions [12]. However, this patient developed symptoms soon after two infusions, or 30 days after starting pembrolizumab. This case highlights an atypical, early presentation of an already rare complication of pembrolizumab.

## Conclusions

CIP is a rare but serious complication of pembrolizumab therapy. While diagnosis can be challenging, it is essential that care teams maintain a high index of suspicion of CIP in patients on pembrolizumab who present with new or worsening respiratory symptoms, regardless of timing. Early recognition and intervention are critical to preventing respiratory decline and improving outcomes.
